# Candidate effectors contribute to race differentiation and virulence of the lentil anthracnose pathogen *Colletotrichum lentis*

**DOI:** 10.1186/s12864-015-1836-2

**Published:** 2015-08-22

**Authors:** Vijai Bhadauria, Ron MacLachlan, Curtis Pozniak, Sabine Banniza

**Affiliations:** Crop Development Centre/Department of Plant Sciences, University of Saskatchewan, Saskatoon, SK S7N 5A8 Canada

**Keywords:** Fungal pathogenicity and virulence, Plant disease resistance, Extended phenotype, *Colletotrichum*

## Abstract

**Background:**

The hemibiotroph *Colletotrichum lentis*, causative agent of anthracnose on *Lens culinaris* (lentil) was recently described as a new species. During its interaction with the host plant, *C. lentis* likely secretes numerous effector proteins, including toxins to alter the plant’s innate immunity, thereby gaining access to the host tissues for nutrition and reproduction.

**Results:**

*In silico* analysis of 2000 ESTs generated from *C. lentis*-infected lentil leaf tissues identified 15 candidate effectors. *In planta* infection stage-specific gene expression waves among candidate effectors were revealed for the appressorial penetration phase, biotrophic phase and necrotrophic phase. No sign of positive selection pressure [ω (dN/dS) < 1] in effectors was detected at the intraspecific level. A single nucleotide polymorphism in the ORF of candidate effector *ClCE6*, used to develop a KASPar marker, differentiated perfectly between pathogenic race 0 and race 1 isolates when tested on 52 isolates arbitrarily selected from a large culture collection representing the western Canadian population of *C. lentis*. Furthermore, an EST encoding argininosuccinate lyase (Arg) was identified as a bacterial gene. A toxin protein ClToxB was further characterized as a potential host-specific toxin through heterologous *in planta* expression. The knock-down of *ClToxB* transcripts by RNAi resulted in reduced virulence, suggesting that *ClToxB* is a virulence factor. *In silico* analysis of the *ClToxB* sequence and comparative genomics revealed that *ToxB* is unlikely a foreign gene in the *C. lentis* genome. Incongruency between established species relationships and that established based on gene sequence data confirmed *ToxB* arose through evolution from a common ancestor, whereas the bacterial gene *Arg* identified in *C. lentis* was horizontally transferred from bacteria.

**Conclusions:**

EST mining and expression profiling revealed a set of *in planta* expressed candidate effectors. We developed a KASPar assay using effector polymorphism to differentiate *C. lentis* races. Comparative genomics revealed a foreign gene encoding a potential virulence factor Arg, which was horizontally transferred from bacteria into the genus *Colletotrichum*. ClToxB is further characterized as a host-specific toxin that is likely to contribute to quantitative differences in virulence between the races 0 and 1.

**Electronic supplementary material:**

The online version of this article (doi:10.1186/s12864-015-1836-2) contains supplementary material, which is available to authorized users.

## Background

*Colletotrichum truncatum* (Schwein.) Andrus & W.D. Moore was originally identified as the causal agent of anthracnose disease on many legume species, including lentil, soybean, faba bean and pea [1]. However, recent evidence suggests that isolates from several of these hosts were misclassified [2, 3], and *Colletotrichum* isolates from lentil (*Lens culinaris* Medik.) were re-classified as *C. lentis* Damm as part of the destructivum clade [4]. This pathogen initiates infection through single-celled conidia that attach to the aerial parts of the host plants, and germinate to form appressoria instrumental in the mechanical breaching of the host surface. Thin penetration pegs arising from beneath the appressoria pierce the host cuticle and cell wall, and differentiate into large bulbous invasive primary hyphae that are biotrophic in nature. Plasmolyzed plant cells colonized by *C. lentis* show a weak interaction between the biotrophic hyphae and the plasma membrane [5]. The primary hyphae of *C. lentis* are entirely confined to the first infected epidermal cells throughout the biotrophic phase. After an initial period of biotrophic infection, the morphogenetic differentiation of thin filamentous necrotrophic secondary hyphae from the thick biotrophic primary hyphae occurs and this transition, referred to as the biotrophy-necrotrophy switch, coincides with large scale cell death and tissue collapse. At this stage, anthracnose lesions become evident on the aerial parts of plants, and thousands of conidia are eventually produced in acervuli developing in these lesions, which reinitiate the disease cycle.

Two pathogenic races were described in the Canadian population of *C. lentis* [6]. Although not characterized by the classical hypersensitive response that limits infection of an avirulent race of a biotrophic pathogen, isolates of the less virulent race 1 of *C. lentis* showed lower conidial germination and formation of appressoria, and differences in the speed and extend of destruction during the necrotrophic phase after inoculation onto a partially resistant lentil cultivar compared to isolates of the more virulent race 0 [7].

Plant pathogenic fungi secrete small proteinaceous and non-proteinaceous molecules in their hosts to manipulate host cell structure and function, thereby facilitating infection (virulence factors and toxins) or triggering host plant defense responses (avirulence factors and elicitors) or both [8–10]. Some of these effectors exert their activity in the apoplast where they may interfere with host plant defense processes, e.g. by inhibiting plant proteases and lytic enzymes. Others are trafficked into host cells, the mechanism of which in terms of traversal of the plasma membrane has recently been disclosed. The role of the amino terminal RxLR and dEER motifs of analogous oomycete effectors in host cell entry has been established [11]. Variants of the RxLR motif have been identified in five fungal effectors (AvrL567, AvrM, Avr2, AvrLm6 and AvrPita), which bind to the phospholipid phosphatidylinositol-3-phosphate located at the exterior leaflet of the plant cell plasma membrane. This binding may facilitate the uptake of effectors through endocytosis [12]. Cytoplasmic effectors can suppress the first layer of inducible defense known as pathogen associated molecular patterns-triggered immunity (PTI) that is activated when conserved pathogen molecules, such as flagellin, EF-Tu, peptidoglycan and chitin are perceived by pathogen recognition receptors located at the cell surface. Suppression occurs by interfering with the signal transduction pathways involved in the manifestation of host defense responses. However, in resistant plants, these cytoplasmic effectors are recognized by disease resistance proteins, most of which possess nucleotide binding and leucine rich repeat domains, thus eliciting effector-triggered immunity (ETI). ETI is an accelerated and amplified PTI response, resulting in disease resistance and usually a localized hypersensitive cell death response (HR) at the infection site [13, 14]. The role of toxins is well documented for necrotrophic pathogens, such as *Stagonospora nordorum* and *Pyrenophora tritici-repentis*. The tan spot pathogen *P. tritici-repentis* delivers two proteinaceous toxins PtrToxA and PtrToxB, and one non-proteinaceous toxin PtrToxC into wheat cells to condition virulence. PtrToxA induces necrosis in wheat genotypes carrying the toxin receptor Tsn1 whereas PtrToxB and PtrToxC elicit chlorosis in different wheat genotypes. Based on their ability to produce these toxins, eight *P. tritici-repentis* races have been described on wheat [15, 16].

During evolution of their genomes, plant pathogenic fungi have also acquired foreign genes through horizontal gene transfer (HGT) from both eukaryotes and prokaryotes. HGT is the nonsexual stable transmission of genetic material between genomes of different species [17, 18], and transferred genes are in most cases involved in the interaction with the host plant [19]. One such example is the transfer of PtrToxA from *S. nodorum* to *P. tritici-repentis* allowing this fungus to become a major pathogen of wheat [20].

Studies conducted to date have identified a handful of candidate effectors from *Colletotrichum* spp. A proline rich glycoprotein, *Colletotrichum* intracellular hyphae 1 (CIH1) was detected in the biotrophic interface of *C. lindemuthianum* and bean cells [21]. The CIH1 peptide sequence contains lysin motifs, which recognize and bind to N-acetyl D-glucosamine, and thus may play a role in protecting fungal chitin from plant chitinases, or in camouflaging the fungus from being detected by the basal defense system [22]. The role of the effector CgDN3 in suppressing HR induced by *C. gloeosporioides* in the tropical pasture legumes *Stylosanthes guianensis* was confirmed by targeted gene disruption. The ∆*CgDN3* mutants elicited a localized HR in host plants, suggesting a role of *CgDN3* in averting HR in susceptible hosts during the biotrophic phase of fungal infection [23]. A yeast signal sequence trap cDNA library was constructed from *in vitro* grown mycelia of *C. graminicola*, the causal agent of stem rot and leaf anthracnose on maize to capture genes encoding secretory proteins, and 103 unique sequences were identified as secretory protein-encoding genes [24]. To identify secretory proteins potentially involved in the virulence of *C. higginsianum* on host plants like *Arabidopsis* and brassicas, a cDNA plasmid library was constructed using RNA isolated from *in vitro* formed appressoria, and fifty-three unique sequences were predicted to encode putative secretory proteins, including 26 secretory proteins lacking a transmembrane domain (extracellular secretory proteins) [25]. Takahara *et al*. [26] developed a fluorescence-activated cell sorting (FACS) method to purify the intracellular biotrophic hyphae from *C. higginsianum*-infected *Arabidopsis* leaves, constructed a biotrophy-specific cDNA library, and identified a set of ESTs encoding putative secretory proteins. We previously constructed a cDNA library from the biotrophy-necrotrophy switch of a *C. lentis* isolate and identified 122 unique sequences encoding potential secretory proteins, including eleven candidate effectors [5]. Among them was the effector protein CtNUDIX, a potential biotrophy-necrotrophy switch regulator, that was expressed precisely before the switch from biotrophy to necrotrophy and induced HR. Overexpression of the *CtNUDIX* in *C. lentis* and the rice blast pathogen *M. oryzae* resulted in incompatibility with their host plants, suggesting a potential role of this biotrophy-necrotrophy switch-specific effector in establishing hemibiotrophy [27]. Deep sequencing of the *C. higginsianum* transcriptome associated with penetrating appressoria, FACS-isolated biotrophic hyphae, and the *in planta* late necrotrophy, yielded 327 unique sequences encoding secreted extracellular proteins. Among these were 198 unigenes encoding *Colletotrichum*-specific effector candidates, of which 102 were absent in the necrotrophy-associated transcriptome, and thus were considered as biotrophy-associated candidate effectors implicated in the establishment of biotrophy (appressorium penetration and development of biotrophic hyphae) [28]. Using fluorescent protein tagging and immunogold transmission electron microscopy, the authors showed a focal secretion of effectors during penetration via appressorium, likely to establish biotrophy. In addition, antagonistic effectors (inducing or suppressing plant cell death) were identified in the study. O’Connell *et al*. [3] analyzed the entire genomes and transcriptomes of *C. higginsianum* and of *C. graminicola*, and concluded that effectors and secondary metabolism enzymes are induced before penetration and during biotrophy, whereas hydrolyzing enzymes and transporters are active during the biotrophy-necrotrophy switch.

The objectives of the present study were to identify candidate effectors among 2000 expressed sequence tags (ESTs) generated from *C. lentis*-infected lentil leaf tissues, understand their expression pattern during pathogenesis and develop Kompetitive Allele Specific PCR (KASPar) markers to differentiate *C. lentis* races. Fifteen candidate effectors putatively secreted by *C. lentis* during the colonization of lentil were identified, and quantitative RT-PCR was performed to profile their expression in an infection time-course. Correlation of expression profiles with race identity was assessed. Homologs were found for 10 of the candidate effectors in other *Colletotrichum* spp. that displayed high peptide sequence identity. We also characterized the toxin gene *ClToxB* through heterologous agroinfiltration in tobacco and RNAi, and showed that it is likely a host-specific toxin and a virulence factor. Homologs were found in five fungal species including three in *Colletotrichum*. While identifying at least one HGT event (*ClArg* encoding argininosuccinate lyase) from bacteria into the genus *Colletotrichum*, comparative genomics analysis revealed that *ClToxB* is a native gene and not horizontally transferred from other species.

## Results

### Identification of *C. lentis* candidate effectors

In a previous study, we constructed a directional cDNA plasmid library from Eston (no resistance to anthracnose) leaf tissues infected with *C. lentis* isolate CT-21 (race 1) undergoing the morphogenetic biotrophy-necrotrophy transition [5]. In the present study, 2000 previously uncharacterized ESTs were sequenced and subjected to BLASTX analysis, and 780 ESTs were identified as ESTs of fungal origin. The remaining ESTs either were of plant origin (~53 %) or unknown (~4 %). The ORF finder, and SignalP and iPSORT algorithms were employed to identify open reading frames (ORFs) containing putative signal peptide (SP) sequences. Twenty-two ORFs were predicted to encode proteins with a putative N-terminal SP for secretion. Out of these 22 ESTs, 13 ESTs represented unique sequences (unigenes) and the remaining 9 ESTs were assembled into two individual unique sequences (Table 1) and were deposited in the NCBI GenBank EST database (dbEST). The average GC content of these unigenes was close to 59 %.

**Table 1:** *Colletotrichum lentis* candidate effectors

Effector	GenBank ID	Peptide (aa)	N/O-Gly	Putative function	Accession	Organism	*E* value
ClCE1	JK998669	189	2/4	Hypothetical protein	EFQ27227	*Glomerella graminicola*	3.00E-53
ClCE2	JK998670	176	1/22	Hypothetical protein	EFQ26411	*Glomerella graminicola*	3.00E-26
ClCE3	JK998671	153	2/5	Hypothetical protein	EFQ27227	*Glomerella graminicola*	7.00E-33
ClCE4	JK998672	236	1/2	Secreted protein	EGG09255	*Melampsora larici-populina*	1.00E-08
ClCE5	JK998673	167	1/1	Collagen-like protein Mcl1	EFY89687	*Metarhizium acridum*	2.00E-14
ClCE6	JK998674	249	1/5	Hypothetical protein	EFQ33016	*Glomerella graminicola*	3.00E-148
ClCE7	JK998675	132	1/5	Hypothetical protein	XP_001912175	*Podospora anserina*	2.00E-12
ClCE8	JK998676	299	1/5	Fasciclin domain protein	EFQ34995	*Glomerella graminicola*	5.00E-85
ClCE9	JK998677	133	1/1	-	-	*Colletotrichum lentis*	-
ClCE10	JK998678	204	0/0	Hypothetical protein	XP_003002566	*Verticillium albo-atrum*	3.00E-44
ClCE11	JK998679	129	0/2	Hypothetical protein	EFQ28429	*Glomerella graminicola*	6.00E-52
ClCE12	JK998680	112	0/0	Hypothetical protein	EFQ27407	*Glomerella graminicola*	1.00E-56
ClCE14	JK998682	158	0/6	Laccase-1 precursor	CBY01468	*Leptosphaeria maculans*	7.00E-04
ClCE15	JK998683	153	1/0	-	-	*Colletotrichum lentis*	-
ClCE18	JK998686	85	0/0	Hypothetical protein	ELA28866	*Colletotrichum gloeosporiodes*	5.00E-20

These effectors were identified from a directional cDNA plasmid library from leaf tissues of *Lens culinaris* cv. Eston infected with *C. lentis* isolate CT-21 undergoing the morphogenetic biotrophy-necrotrophy transition

The translated amino acid sequences of the putative secretory proteins were analyzed for features indicative of secretory proteins that are likely to enter into host cells. No transmembrane helices were detected in these proteins, and we considered them soluble secretory proteins (referred to hereafter as candidate effectors). However, N- and O-glycosylation sites were predicted in the peptide sequences of 13 candidate effectors indicating potential for their attachment to the fungal cell membranes and cell walls. The N- and O-glycosylation sites allow for attachment of sugar chains to asparagine residues, and serine and threonine residues, respectively.

The BLASTP algorithm was used to identify putative functions of these candidate effectors. Of 15 candidate effectors, ten (ClCE1 through 4, ClCE6, ClCE7, ClCE10 through 12, ClCE18) were identified as hypothetical proteins (Table 1). Two candidate effectors (ClCE9 and ClCE15) had no significant BLASTP match at a cut-off of *E* value ≤1e-6. These were queried with TBLASTN against 68,986 unigenes derived from 22 fungal and oomycete species in the COGEME EST database, and finally considered as *C. lentis* orphan sequences due to lack of significant matching hits. However, both candidate effectors were perfectly mapped onto the recently assembled *C. lentis* isolate CT-30 (race 0) draft genome (unpublished data). The remaining three showed significant similarity to collagen-like protein Mcl1 (ClCE5), fasciclin domain-containing protein (ClCE8) and laccase-1 precursor (ClCE14) with *E* value ≤ 1e-6. Mcl1 (*Metarhizium* collagen-like protein 1) acts as an antiadhesive protective coat by camouflaging antigenic structural components of the cell wall, such as β-glucans [29]. Fungal fasciclin domain-containing proteins are cell surface proteins and known to attach to the exterior leaflet of the plasma membrane by glycosylphosphatidylinositol (GPI) anchors [30]; however, ClCE8 lacks a GPI anchor addition site in its predicted peptide sequence. Laccases (EC 1.10.3.2, p-diphenol: dioxygen oxidoreductase) are copper-oxidoreductases that catalyze the biological oxidation-reduction of polyphenols with a concomitant reduction of molecular oxygen to water [31].

### Candidate effectors of *C. lentis* show striking similarity to those of other *Colletotrichum* species

**Fig. 1:**
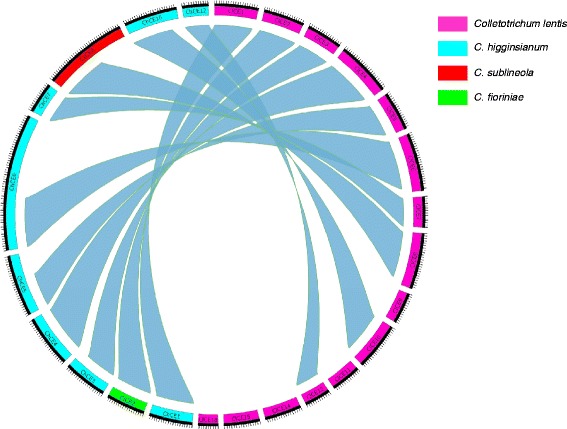
Comparative analysis of *Colletotrichum lentis* candidate effectors with other *Colletotrichum* spp. The Circos plot shows candidate effectors as ideograms. Stringent *E* value 1e-50 was used to identify potential homologs and only top BLAST hit was included in the Circos plot. Ribbon links convey the linked region between two ideograms with over 65 % amino acid residue identities

Effectors have long been regarded as a relatively species-specific repertoire of arms that dismantles resistance evolved or introgressed in host plant species [32], hence the likelihood of finding homologs in other species has been considered low. Exploiting the availability of an increasing number of fungal genomes, putative effectors were mined here at a global scale in *C. lentis* and other species with particular emphasis on the four available genomes in the genus *Colletotrichum* to identify and compare homologs to *C. lentis* putative effectors. With a stringent *E* value of 1e-50, we identified potential homologs of 10 out of 15 *C. lentis* candidate effectors. Top hits were found in *Colletotrichum* spp. and used in comparative pairwise sequence analysis. A Circos plot [33] depicting 25 ideograms representing 15 candidate effectors from *C. lentis* and 10 potential homologs from *C. higginsianum*, *C. sublineola* and C. *fioriniae* was generated to visualize the percentage peptide sequence similarities between candidate effectors and their homologs (Fig. 1). Covering over 80 % of the effector sequences, these homologs show more than 65 % peptide sequence identity, suggesting that effectors of different, but related species are not as unique as previously thought and can display significant homology across species.

### Time-course expression profiling of candidate effectors

Using RT-qPCR, expression of candidate effectors *in planta* was quantified at appressorium penetration (24 hai), during the biotrophic (44 hai) and necrotrophic phase (72 hai), as well as in mycelia and ungerminated conidia. The transcription levels of candidate effectors were normalized to the *C. lentis* house-keeping gene actin, and were expressed as relative values with 1 corresponding to expression level in mycelia.

**Fig. 2:**
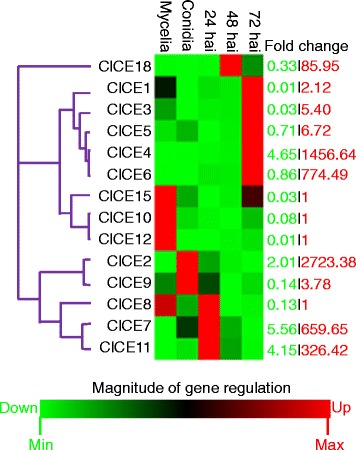
Clustergram of the *Colletotrichum lentis* candidate effector expression profiles. Each column represents either an *in vitro* cell type or an *in planta* (lentil) infection time-point and each row represents an effector gene. Red and green colors in rows indicate up- and down- regulation in the gene expression, respectively. The fold change column indicates minimum (green) and maximum (red) fold change values. Hai, hours after inoculation

Nine out of 14 candidate effector genes showed upregulation in their expression during *in planta* infection whereas the remaining five genes were repressed during plant infection (Fig. 2). The C_T_ value for the effector gene *ClCE14* was higher (C_T_ >35) and therefore was not further analyzed. Three expression waves were noticeable among genes induced during infection: Appressorium penetration-specific (*ClCE7, ClCE8* and *ClCE11*), biotrophy-specific (*ClCE18*) and necrotrophy-specific (*ClCE1* and *ClCE3* through *6*). Two candidate effectors (*ClCE2* and *ClCE9*) showed conidia-specific expression.

### Effector polymorphism in *C. lentis* isolates collected from western Canada

**Fig. 3:**
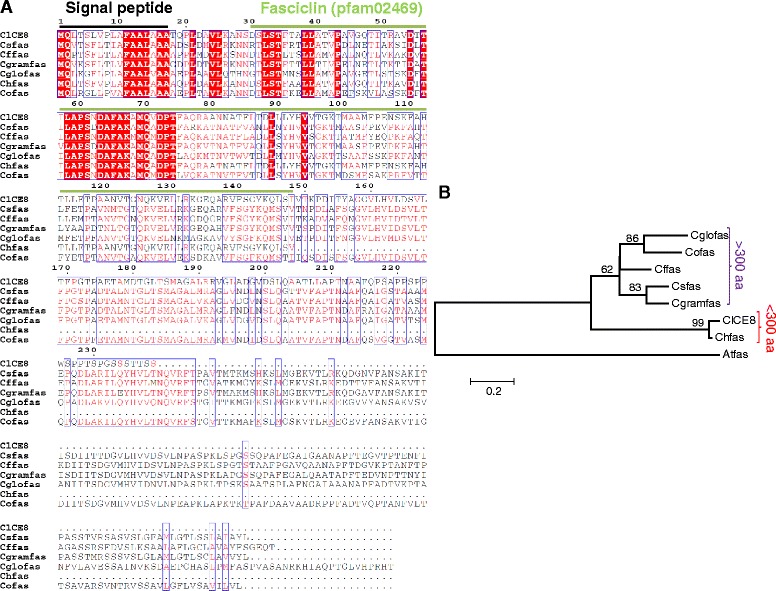
*Colletotrichum lentis* ClCE8 homologs are conserved in fungal pathogens. **a**. Six ClCE8 (JK998676) homologs are identified in the NCBI non-redundant protein database using a stringent *E* value (1e-50). Csfas, *Colletotrichum sublineola* (KDN70693); Cffas, *Colletotrichum fioriniae* (XP_007601018); Cgramfas, *Colletotrichum graminicola* (EFQ34995); Cglofas, *Colletotrichum gloeosporioides* (EQB59239); Chfas, *Colletotrichum higginsianum* (CCF47579); and Cofas, *Colletotrichum orbiculare* (ENH86023). Peptide sequences were aligned using ESPrint version 3.0. Putative signal peptides (signature characteristic of effectors) were predicted using SignalP version 4.1. Signal peptide and fasciclin domains (pfam02469) are over-lined with black and green colors, respectively. **b**. A Neighbor-Joining phylogram of ClCE8 homologs. The *Arabidopsis thaliana* fasciclin-like arabinogalactan protein (Atfas, NP_566398) was used as an outgroup. The evolutionary distance was estimated using a Poisson-correction model and is in the units of the number of amino acid substitutions per site. Values at nodes indicate the percentage of branch support derived from 1000 bootstrap replicates

Considering the hyper-variable nature of effectors, we scanned all 15 candidate effectors against the draft genome of *C. lentis* isolate CT-30 (race 0) and identified two silent SNPs (Race 1/Race 0, T/C) in the two candidate effectors *ClCE6* and *ClCE8*. ClCE6 is a hypothetical protein whereas ClCE8 contains a fasciclin domain (pfam02469). With a stringent *E* value of 1e-50, six homologs of ClCE8 belonging to *C. sublineola*, *C. fioriniae*, *C. graminicola, C. gloeosporioides*, *C. higginsianum* and *C. orbiculare* were identified in the NCBInr protein database. All six homologs contain a putative 17-aa SP for potential secretion in host plants and a fasciclin domain (variable in length) for function. ClCE8 homologs are relatively large proteins (>300 aa) except for the *C. higginsianum* fasciclin protein (Chfas [CCF47579], 148-aa), suggesting ClCE8 and Chfas are likely splicing variants. ClCE8 homologs contain conserved amino acid residue blocks (in red in Fig. 3a) in the fasciclin domain, indicating functional homology among homologs, and most variability is seen at the C-terminus. Fungal fasciclin proteins are known to function as virulence factors [34].

ClCE8 homologs were retrieved from the NCBInr protein database using a stringent *E* value of 1e-50. A Neighbor-Joining phylogram of ClCE8 homologs was generated using a Poisson-correction model (K = −ln(1-D)). ClCE8 and Chfas from *C. higginsianum* formed a cluster separate from the remaining six fasciclin proteins with lowest mean number of amino acid substitution(s) per site (K = 0.02) between them, suggesting that both putative effectors are likely functional homologs (Fig. 3b). Considering the small size (<300 aa), both ClCE8 and Chfas are likely delivered in host plants by their respective pathogens to facilitate fungal colonization.

**Fig. 4:**
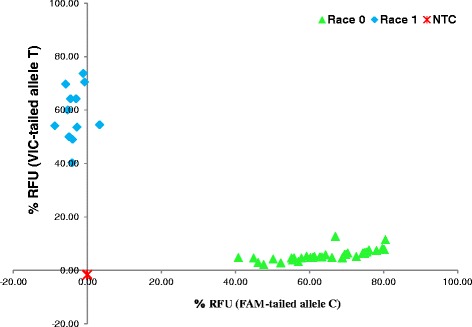
Allele discrimination plot based on percentage endpoint fluorescence (RFU) in KASPar assays. Fifty two *Colletotrichum lentis* isolates collected from western Canada were genotyped using the *ClCE6* KASPar marker. NTC: No Template Control

**Fig. 5:**
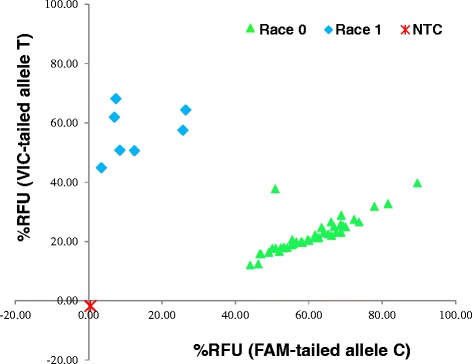
Allele discrimination plot based on percentage endpoint fluorescence (RFU) in KASPar assays. Fifty two *Colletotrichum lentis* isolates collected from western Canada were genotyped using *ClCE8* KASPar marker. NTC: No Template Control

To test whether SNPs in *ClCE6* and *ClCE8* can differentiate virulent race 0 from less virulent race 1 isolates, 52 *C. lentis* isolates collected from western Canada were randomly selected and phenotyped using the lentil differential cultivar CDC Robin with partial resistance to race 1, but not to race 0 isolates. Thirty nine out of 52 isolates were fully pathogenic on CDC Robin and therefore classified as race 0 isolates. The remaining 13 isolates caused significantly fewer lesions on the cultivar, hence were classified as race 1 isolates. KASPar assays revealed that the *ClCE6* KASPar marker could differentiate race 0 from race 1 isolates based on a correlation of 100 % between genotypic and phenotypic assay results. In contrast, the *ClCE8* KASPar marker identified six race 1 isolates as race 0. Results suggest that *ClCE6* is likely co-segregated with loci governing the virulence of *C. lentis* (Table 2, Figs. 4 and 5).

**Table 2:** Race indexing of *Colletotrichum lentis* isolates based on phenotyping and genotyping

Isolates	Race indexing		
	Phenotype	ClCE6	ClCE8
Cl-11	0	0	0
Cl-15	1	1	1
Cl-16	0	0	0
Cl-17	1	1	1
Cl-20	0	0	0
Cl-21	1	1	1
Cl-23	0	0	0
Cl-26	0	0	0
Cl-28	1	1	1
Cl-29	1	1	1
Cl-30	0	0	0
Cl-31	0	0	0
Cl-32	0	0	0
Cl-33	1	1	0
Cl-34	0	0	0
Cl-35	1	1	0
Cl-37	0	0	0
Cl-38	0	0	0
Cl-39	1	1	0
Cl-43	1	1	1
Cl-44	0	0	0
Cl-45	0	0	0
Cl-46	0	0	0
Cl-47	0	0	0
Cl-58	1	1	1
Cl-59	0	0	0
Cl-60	0	0	0
Cl-181	0	0	0
Cl-185	0	0	0
Cl-187	0	0	0
Cl-188	0	0	0
Cl-189	0	0	0
Cl-190	0	0	0
Cl-191	0	0	0
Cl-192	0	0	0
Cl-209	0	0	0
Cl-231	0	0	0
Cl-233	0	0	0
Cl-255	0	0	0
Cl-364	0	0	0
Cl-366	0	0	0
Cl-368	0	0	0
Cl-379	0	0	0
Cl-380	0	0	0
Cl-383	0	0	0
Cl-384	1	1	0
Cl-397	0	0	0
Cl-400	1	1	0
Cl-402	1	1	0
Cl-417	0	0	0
Cl-418	0	0	0
Cl-422	0	0	0

Differential *Lens culinaris* cv. CDC Robin, and *ClCE6* and *ClCE8* KASPar markers were used to phenotype and genotype *C. lentis* isolates, respectively

### *ClToxB* is likely a host-specific toxin and implicated in virulence differentiation of *C. lentis* races

**Fig. 6:**
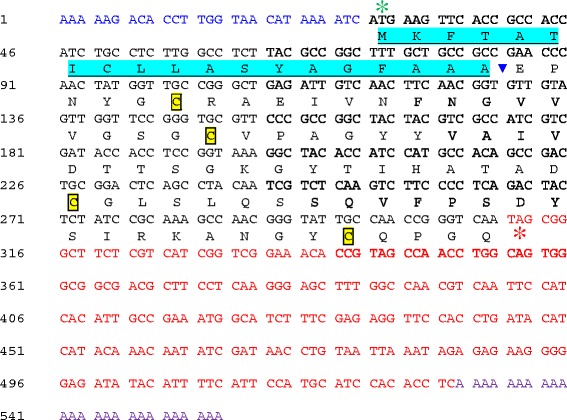
*Colletotrichum lentis ClToxB* cDNA sequence. Green and red asterisks indicate start and stop codons. Signal peptide for potential secretion is highlighted in turquoise color. Cysteine residues are highlighted and boxed

**Fig. 7:**
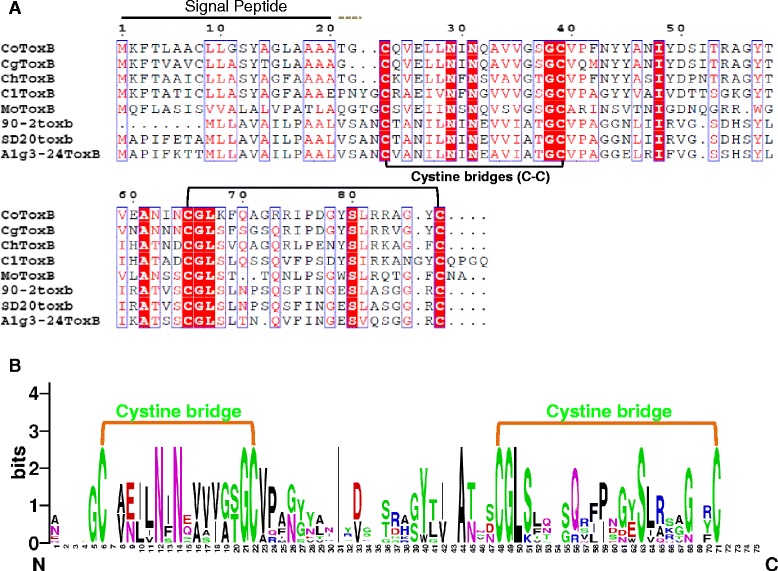
Structural similarity of *Colletotrichum lentis* ClToxB with ToxB of other species. **a**. ClToxB shows structural similarity to ToxB proteins from closely related species and 3 ToxB variants from *Pyrenophora tritici-repentis*. Four conserved cysteine residues were identified in the mature protein, and these amino acid residues are likely to form two cystine bridges, which may provide stability to ToxB proteins in the host cell apoplast. Peptide sequences were aligned using ESPrint version 3.0. Putative signal peptides (black line) were predicted using SignalP version 4.1. ClToxB, *Colletotrichum lentis* (Race 1, CT-21 [less virulent isolate] and Race 0, CT-30 [virulent isolate]); CoToxB*, C. orbiculare* 104-T (virulent isolate); CgToxB, *C. gloeosporiodes* Nara gc5 (virulent isolate); ChToxB, *Colletotrichum higginsianum* IMI349063 (virulent isolate); MoToxB, *Magnaporthe oryzae* Y34 (virulent isolate); 90-2toxb, *Pyrenophora tritici-repentis* 90–2 (Race 4, 90–2 [avirulent isolate]); SD20toxb, *Pyrenophora tritici-repentis* (Race 4, SD20 [avirulent isolate]); and Alg3-24ToxB, *Pyrenophora tritici-repentis* (Race 5, Alg3-24 [most virulent isolate]). **b.** ClToxB homologs contain a conserved cysteine residue pattern. Sequence logo was generated from the Clustal W-aligned ToxB mature proteins using Seg2Logo (Thomsen and Nielsen [50]). Big and small amino acid residue stacks indicate conserved and variable sites, respectively. Conserved cysteine residues are likely to form two cystine bridges (disulfide bonds)

**Fig. 8:**
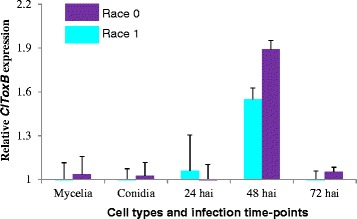
Comparative expression profiling of *Colletotrichum lentis ClToxB*. X-axis shows two fungal cell types (mycelia and conidia) and three *in planta* infection time-points (24 hai, 48 hai and 72 hai) whereas Y-axis shows relative gene expression on a log2 scale. Hai: Hours after inoculation

In a previous study on EST mining of *C. lentis* infected lentil tissues, *ClToxB* (GenBank accession JZ350031) was identified [5]. Unlike *PtrToxB* of *P. tritici-repentis*, only one copy of *ClToxB* was detected in the *C. lentis* genome (unpublished data). The full length cDNA of *ClToxB* was sequenced here revealing an open reading frame of 282-bp with a 27-bp 5-prime untranslated region (UTR) and a 218-bp 3-prime UTR that encodes a pre-protein of 94-aa. An SP of 19-aa with a cleavage site in-between alanine-19 and glutamate-20 was predicted in the pre-protein, and four cysteine residues were found in the mature protein (75-aa) (Fig. 6). ClToxB is a small (94 aa), stable (instability index 20.99), soluble (lacks transmembrane helix, N/O-glycosylation and GPI addition sites) and acidic protein (p*I* 5.52) protein with 7.85 KDa molecular weight (Table 3). Using the non-redundant protein database available at the NCBI with an *E* value cut-off of 1e-6, homologs of ClToxB were identified in *C. higginsianum*, *C. orbiculare*, *C. gloeosporiodes*, and *Magnaporthe oryzae*. Multiple sequence alignment with ClustalW revealed extensive similarity with PtrToxB including that of the most virulent isolate alg3-24 (Fig. 7a, Table 4**)**. ClToxB is a conserved protein with 4 characteristic cysteine residues. Using the DiANNA web server, two disulfide bonds were predicted in the mature protein, which may provide stability to ClToxB in the host apoplast (Fig. 7b), and RT-qPCR analysis showed that the expression of *ClToxB* peaked during the biotrophy-necrotrophy switch (48 hpi), suggesting a potential role in anthracnose development. Expression polymorphism was detected at 48 hpi as the *ClToxB* transcript level was higher in lentil tissues infected with the more virulent isolate CT-30 (race 0) than that inoculated with CT-21 (race 1) (Fig. 8). This differential transcript level may be associated with virulence patterns of *C. lentis* races. To investigate whether *ClToxB* also shows sequence polymorphism, the full length cDNA from isolate CT-21 (race 1) was mapped onto the CT-30 (race 0) draft genome. No DNA polymorphism was detected between the two isolates representing races 1 and 0.

**Table 3:** ClToxB biochemical properties predicted through *in silico* analyses

Characteristics	Value	Softwares
Signal peptide	1	SignalP v4.1 & iPSORT
Cysteine residues	4	Mannual counting
Cystine bridges	2	DiANNA v1.1
Isoelectric point (pI)	5.52	ExPASy Compute PI/Mw
Molecular weight	7.85 KDa	ExPASy Compute PI/Mw
Transmembrane helix	0	TMHMM v2.0
GPI addition site	0	Big-PI Fungal Predictor v3.0
N-glycosylation site(s)	0	NetNGlyc 1.0
O-glycosylation site(s)	0	NetOGlyc v3.1
Grand average of hydropathicity	-0.087	ProtParam
Instability index	20.99	ProtParam

**Table 4:** ClToxB homologs

Toxin B homologs	Pathogen	GenBank	Isolate/Strain	Peptide (aa)	SP^a^	MP^b^	Identity |Similarity^c^
ClToxB	*Colletotrichum lentis*	JZ350031	CT-21 (Race 1) & CT-30 (Race 0)	94	19	75	100 | 100
Ptrtoxb	*Pyrenophora tritici-repentis*	AAM00019	SD20 (Race 4)	88	23	65	40 | 50
PtrToxB	*Pyrenophora tritici-repentis*	AF483831	Alg3-24 (Race 5)	87	23	64	39 | 53
CgToxB	*Colletotrichum gloeosporiodes*	ELA28482	Nara gc5	86	19	67	44 | 57
ChToxB	*Colletotrichm higginsianum*	CCF45936	IMI349063	87	19	68	52 | 66
CoToxB	*Colletotrichum orbiculare*	ENH84621	104-T	87	19	68	44 | 58
MoToxB	*Magnaporthe oryzae*	ELQ42910	Y34	88	20	68	36 | 52
Ptrtoxb	*Pyrenophora tritici-repentis*	AF483832	90-2 (Race 4)	80	15	65	40 | 50

An *E* value cut-off of 1e-6 was used to extract homologs of ClToxB from NCBInr protein database. All homologs contain four characteristic cystine residues, which are likely to form 2 disulfide bonds. ^a^SP, Signal Peptide (predicted using SignalP server version 4.1)^b^MP, Mature Protein^c^Identity/Similarity (in per cent) as calculated by Needleman-Wunsch pairwise alignment of ClToxB (MP) with its homologs.

**Fig. 9:**
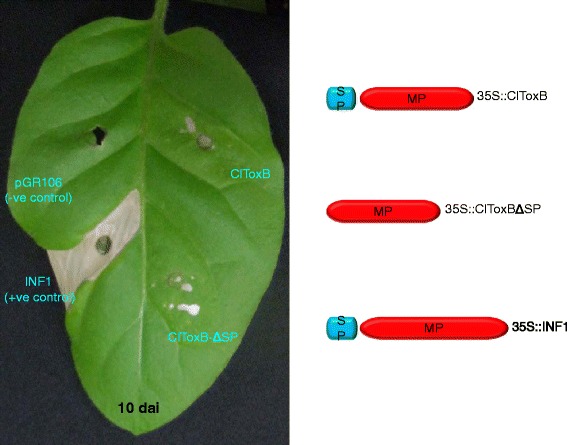
ClToxB is likely a host-specific toxin. *ClToxB* with and without signal peptide were cloned in the PVX-based binary vector pGR106. *Agrobacterium tumefaciens* strains carrying pGR106-ClToxB and pGR106-ClToxBΔSP were expressed in the model plant tobacco (*Nicotiana tobacum*), and macroscopic cell death/necrosis was monitored 3 through 10 days post-infiltration (dpi). *Phytophthora infestans* infestin 1 (INF1) elicitin and empty vector were used as positive and negative controls, respectively. SP, signal peptide and MP, mature protein

To confirm whether ClToxB is a host-specific toxin, *ClToxB* with and without SP were expressed in tobacco via agroinfiltration. *Phytophthora infestans* infestin 1 [35] used as a positive control in the transient expression assay caused confluent cell death at 3 days post-infiltration (dpi) whereas that was not the case for ClToxB though scattered cell death flecks were found in the vicinity of the agroinfiltration site. No chlorosis was observed in the zones infiltrated with ClToxB and ClToxB∆SP, suggesting that ClToxB is likely a host-specific toxin (Fig. 9).

Taken together, data indicate that unlike in the case of *PtrToxB* in *P. tritici-repentis*, presence or absence of *ClToxB* or sequence polymorphisms do not determine the races that have been described in *C. lentis*. However, *ClToxB* may contribute to the virulence profile of races on lentil through differences in the level of expression, thereby amplifying cell death signals at the biotrophy-necrotrophy switch.

### *ClToxB* is unlikely a foreign gene in the *C. lentis* genome

**Fig. 10:**
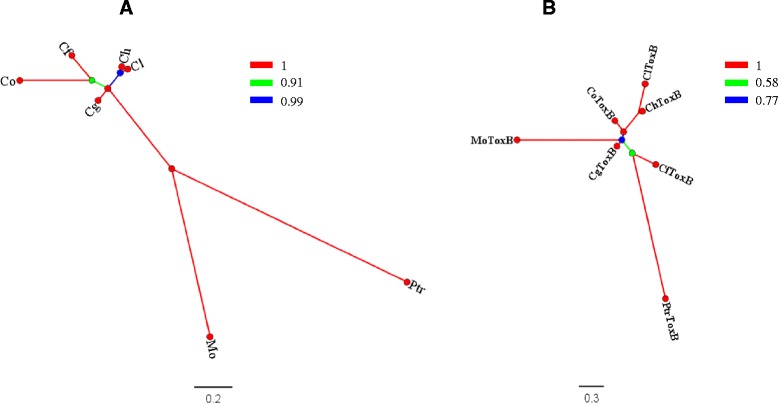
Phylogenetic analysis. **a** Species tree based on the multi-locus concatenated DNA sequence data (ITS-5.8S, GAPDH, CHS-1, HIS3, ACT and TUB2) obtained from heuristic search of sequence data of species. **b** Gene tree based on *ToxB* homologs. Both trees were generated using Bayesian inference algorithm (nucleotide substitution model GTR + G + I). Branch colors and scale bars represent posterior probabilities and evolutionary distance in substitutions per site, respectively. Cl, *Colletotrichum lentis*; Ch, *Colletotrichum higginsianum*; Co, *C. orbiculare*; Cg, *C. gloeosporiodes*; Cf, *C. fioriniae*; Mo, *Magnaporthe oryzae*; and Ptr, *Pyrenophora tritici-repentis*

**Fig. 11:**
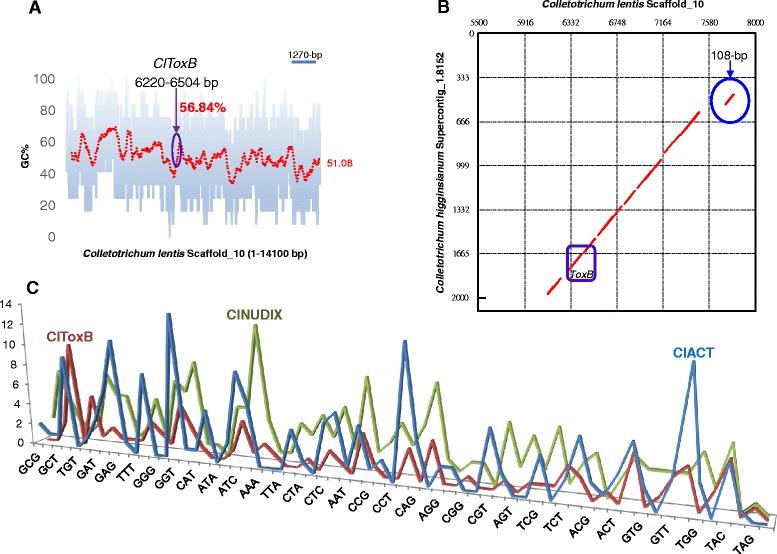
Assessment of the *ClToxB* sequence for evidence of horizontal gene transfer. **a** GC content along the *Colletotrichum lentis* scaffold_10 (14100 bp) containing *ClToxB* gene. **b** MUMmerplot (dot plot) represents NUCmer alignment of *ClToxB* and *ChToxB* loci. X and Y-axes show *C. lentis* scaffold_10 (5500–8000 bp) and *C. higginsianum* supercontig_1.8152 (2079 bp), respectively. Red diagonal line describes the syntenic relationship between *C. lentis* and *C. higginsianum. ToxB* homologs from both species (shown in box) are detected in the conserved syntenic block. A 108-bp translocation (detached line) is circled. **c** Codon usage profiles of *ClToxB*, *ClACT* and *ClNUDIX*. X and Y-axes show 64 codons and their frequencies in sequences, respectively

Comparison of molecular tree topologies generated based on multiple loci (species tree) (Fig. 10a) and based on the sequences of *ToxB* homologs (Fig. 10b) revealed incongruency between the trees, which would be expected in case of HGT. No patchy phyletic distribution of *ToxB* was found in the gene tree, and *ToxB* genes from closely related species like *Colletotrichum* spp. were grouped together as a clade. A 14100-bp fragment containing the *ClToxB* locus (Scaffold_10) retrieved from our newly assembled *C. lentis* draft genome revealed an average of 51.08 and 56.84 GC % for the scaffold and the *ClToxB* gene, respectively, which is not atypical as would be expected in cases of HGTs (Fig. 11a). Genes involved in HGTs are unlikely to be present in closely related species, and if present, do not show syntenic relationship as events of horizontally transferred genes are random rather than targeted. To confirm this hypothesis, a MUMmerplot was generated using NUCmer alignment of the *ToxB* loci contained in Scaffold_10 (5500–8000 bp) from *C. lentis* and Supercontig_1.8152 (1–2079 bp) from *C. higginsianum. ClToxB* and *ChToxB* were located in the conserved syntenic block (Fig. 11b), indicating that *ClToxB* was not acquired by HGT. Recently transferred foreign genes, especially those from prokaryotes, show different preferential codon usage pattern compared to the native genes as they have yet to adapt to their new recipient host genomes. Comparison of the codon usage profile of *ClToxB* with the native house-keeping gene actin (*ClACT*) and a previously characterized biotrophy-necrotrophy switch regulator *ClNUDIX* [27] showed no atypical variation in profiles (Fig. 11c). Taken together, our data confirm that *ClToxB* is a native rather than foreign gene in the *C. lentis* genome.

### *ClToxB* is a virulence factor

**Fig. 12:**
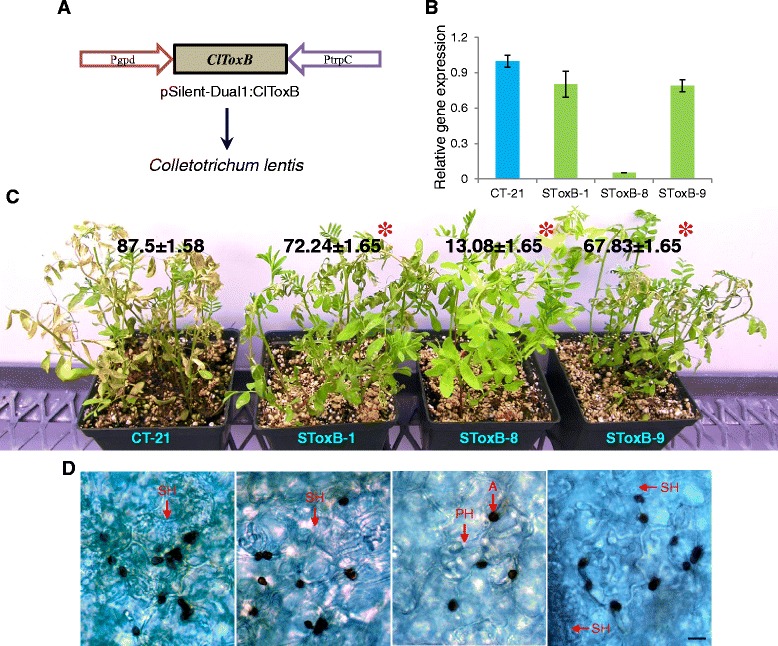
*ClToxB* silencing. **a** RNA-silencing vector pSilent-Dual 1 (Nguyen et al. [55]). It contains two convergent promoters (Pgpd and PtrpC) of *Aspergillus nidulans. ClToxB* (JZ350031) was cloned in-between Pgpd and PtrpC promoters of pSilent-Dual 1, which was then used to transform *Colletotrichum lentis* isolate CT-21. **b** *ClToxB* expression in the resulting transformants (SToxB-1, SToxB-8 and SToxB-9) was determined by RT-qPCR. SToxB-8 displayed only 5 % of the *ClToxB* expression. **c** Susceptible lentil cultivar Eston was used to determine the anthracnose causing ability of silenced strains. Quantitative difference in virulence on Eston was observed among silenced strains at 6 days after inoculation (dai) and was clearly evident in the SToxB-infected plants. Disease scores were reported as least square means ± standard error. **d** Infected tissues collected at 3 dai were visualized under a light microscope. Quantitative difference in virulence was associated with the *in planta* fungal proliferation of silenced strains and the *ClToxB* expression level. A, Appressorium; PH, Primary biotrophic hyphae; and SH, Secondary necrotrophic hyphae. Bar =20 μM

An RNA-silencing approach was used to determine the functional role of *ToxB* in *C. lentis*. The *ClToxB* was cloned into a dual promoter silencing vector pSilent-Dual 1 to generate sense and antisense *ClToxB* RNA pools. The vector pSilent-Dual 1-ClToxB was then used to transform *C. lentis* spheroplasts (Fig. 12a). Fifteen transformants were retrieved from this transformation and subjected to RT-qPCR. No knock down-penalty was observed among silenced strains as they grew normally and conidiogenesis was similar to the wild-type isolate CT-21. Three silenced strains were selected for virulence/pathogenicity testing. Among them was the strain SToxB-8, which displayed only 5 % of the wild-type *ClToxB* expression (Fig. 12b). Susceptible lentil cultivar Eston was used to evaluate the virulence of silenced strains. Lentil plants infected with the silenced strain SToxB-8 showed significantly reduced anthracnose symptoms (disease severity ± SE of 13.08 ± 1.65) compared to the wild-type strain CT-21 (disease severity ± SE of 87.5 ± 1.58) (Fig. 12c). To investigate which stage of fungal development was impaired by the silencing of *ClToxB*, infected leaf tissues were collected at 3 days after inoculation (dai) and visualized under a light microscope. Quantitative variation in virulence was observed during *in planta* fungal proliferation. The silenced strain SToxB with the lowest level of *ClToxB* expression caused fewer anthracnose lesions at 6 dai (Fig. 12c), which was likely associated with the delayed biotrophy-necrotrophy switch as can be seen in case of the SToxB-8 at 3 dai (Fig. 12d).

### *ClArg* is a foreign gene in the genus *Colletotrichum*

**Fig. 13:**
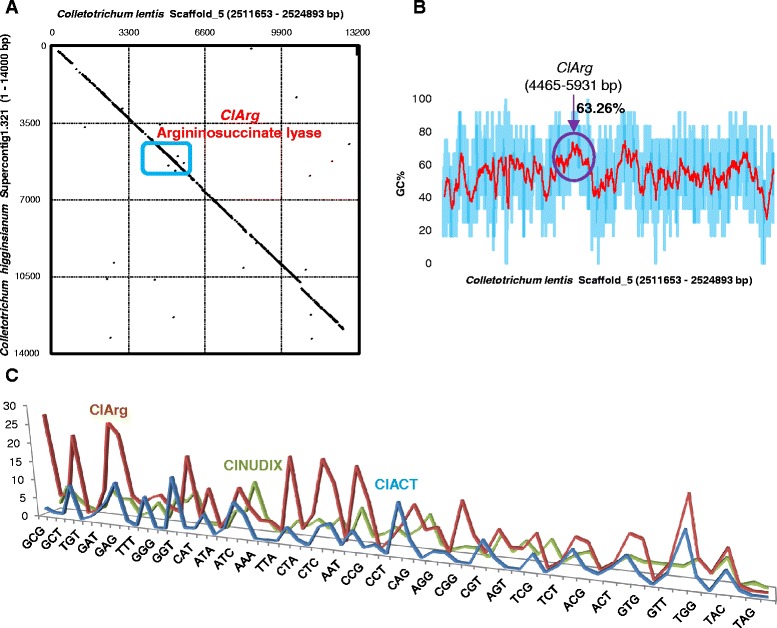
Horizontal gene transfer from bacteria into the genus *Colletotrichum*. **a** MUMmerplot (dot plot) represents NUCmer alignment of *ClArg* and *ChArg* locus-containing scaffolds. X and Y-axes show *C. lentis* scaffold_5 (2511653–2524893 bp) and *C. higginsianum* supercontig_1.321 (14000 bp), respectively. Black diagonal line describes the syntenic relationship between *C. lentis* and *C. higginsianum. ClArg* homologs from both species (shown in box) are detected in the conserved syntenic block. **b** GC content along the *C. lentis* scaffold _5 (13260 bp) containing *ClArg* gene. **c** Codon usage profiles of *ClArg*, *ClACT* and *ClNUDIX*. X and Y-axes show 64 codons and their frequencies in sequences, respectively

ESTs were further scanned for potential (inter kingdom) HGT events using the BLASTX algorithm on Linux standalone BLAST and NCBInr protein database. We hypothesized that the top hits of a HGT candidate with an *E* value 1e-50 should match to distantly related fungal species (in case of intra-kingdom HGT) or bacterial species (in case of inter-kingdom HGT). Only one EST met this criterion and the full length coding sequence (CDS) was retrieved by mapping the EST onto the *C. lentis* draft genome. When the CDS was queried against NCBInr protein database with an *E* value 1e-50 and maximum target sequences 100, the top seven hits were matched to six *Colletotrichum* spp. (with an *E* value 0 and 95–99 % query coverage) and the remaining 93 hits were matched to the kingdom Prokaryota/Bacteria (68 hits with an *E* value 0 and 89–97 % query coverage). The top ten hits are listed in the Table 5. All hits were identified as argininosuccinate lyase (Arg; EC 4.3.2.1) that is implicated in the urea cycle. Arg catalyzes a reaction that forms fumarate and arginine from L-argininosuccinate. *ClArg* is located in a block on scaffold_5 of the *C. lentis* draft genome that is syntenic with a block on supercontig_1.321 of the closely related species *C. higginsianum* (Fig. 13a), suggesting that *Arg* was horizontally transferred into a common ancestor of the genus *Colletotrichum. ClArg* contains no intron, and has a relatively higher GC content (63.25 %, Fig. 13b) compared to the rest of the genome, and atypical codon usage compared to eukaryotic genes *ClACT* and *ClNUDIX* (Fig. 13c), further confirming that *ClArg* is not a native gene in the *C. lenis* genome.

**Table 5:** ClArg homologs

Top 10 hits	Accession	Length (aa)	Putative function	Organism	Query coverage (%)	*E* value	Taxonomic classification	EC code
1	ClArg	488	Argininosuccinate lyase	*Colletotrichum lentis*	99	-	Fungus	EC 4.3.2.1
2	CCF37911	488	Argininosuccinate lyase	*Colletotrichum higginsianum*	99	0	Fungus	EC 4.3.2.1
3	KDN68815	488	Argininosuccinate lyase	*Colletotrichum sublineola*	99	0	Fungus	EC 4.3.2.1
4	EFQ25990	488	Argininosuccinate lyase	*Colletotrichum graminicola*	99	0	Fungus	EC 4.3.2.1
5	XP_007286426	487	Argininosuccinate lyase	*Colletotrichum gloeosporioides* ^a^	99	0	Fungus	EC 4.3.2.1
6	XP_007595184	488	Argininosuccinate lyase	*Colletotrichum fioriniae*	99	0	Fungus	EC 4.3.2.1
7	ENH83872	487	Argininosuccinate lyase	*Colletotrichum orbiculare*	99	0	Fungus	EC 4.3.2.1
8	WP_043836904	504	Argininosuccinate lyase	*Roseomonas aerilata*	96	0	Bacterium	EC 4.3.2.1
9	WP_003075088	522	Argininosuccinate lyase	*Comamonas testosteroni*	96	0	Bacterium	EC 4.3.2.1
10	WP_043360792	504	Argininosuccinate lyase	*Belnapia sp.* F-4-1	96	0	Bacterium	EC 4.3.2.1

Top ten hits were obtained by BLASTing peptide sequence of *Colletotrichum lentis* argininosuccinate lyase against NCBInr protein database

^a^Two hits were matched to two strains of *C. gloeosporiodes.* Only one is listed in the table

## Discussion

Expression of pathogen effectors in plants is a perfect example of the “extended phenotype”, a concept put forward by Richard Dawkins [36] in his classic book “Extended phenotype: The long reach of genes”. Effector biology of filamentous fungi pathogenic to economically important crops is an established field in plant pathology. Since the introduction of next-generation sequencing technologies, the number of sequenced fungal genomes and transcriptomes has been increasing exponentially, and as a result, the discovery of effector genes is accelerating. Effector biology has practical implications for resistance breeding via effector-assisted selection of plants in populations segregating for disease resistance [37–39]. The role of effectors in fungal pathogenicity and virulence is well established, and thus monitoring allelic diversity of effectors in evolving pathogen population and among races can assist in *R* gene deployment in cultivated varieties [38, 39]. Therefore, generating an inventory of candidate effector genes is the first step towards their potential usage in disease resistance breeding in economically important food crops, such as cereals, oilseeds and legumes. Comparison of effector gene structure and function among closely and more distantly related species, now increasingly more feasible as the number of sequenced genomes is increasing rapidly, will also shed new light on the effects of selection on population divergence and speciation, as well as the frequency of HGT events.

We identified 15 candidate effectors of *C. lentis* mined from the biotrophy-necrotrophy switch-specific cDNA library developed previously with the objective to identify effectors that may be involved in the virulence of *C. lentis* on its host lentil, and may differentiate between the two pathogenic races described in the western Canadian population of this pathogen. Among them were two *C. lentis* - specific effectors (*ClCE9* and *ClCE15*). For the majority of candidate effectors (8), the top BLAST hit (*E* value ≤1e-6) was matched to fungal proteins within the genus *Colletotrichum* predicted from automated whole-genome sequencing and annotation projects (Fig. 1). Only three candidate effectors had detectable homologs with putative functions. The majority of identified candidate effectors (8) are *in planta*-induced, but during different stages of lentil infection, namely appressorium penetration, biotrophic and necrotrophic phases. The expression of the remaining candidate effectors displayed no significant alternation between *in vitro* vegetative growth and *in planta* colonization (Fig. 2). This suggests that *C. lentis* deploys different sets of effector proteins to condition and promote virulence.

Putative functions were assigned to 3 candidate effectors based on orthology to proteins of known functions (Table 1). Effector *ClCE5*, a *Mcl1* homolog showed a 6.7-fold induction during the necrotrophic phase (Fig. 2). Mcl1 was originally identified in the entomopathogenic fungus *Metarhizium anisopliae* and was shown to be expressed within 20 min of contact between the pathogen and the hemolymph of the lepidopteran model insect *Manduca sexta*. Mcl1 envelopes *M. anisopliae* hyphae, and in doing so, camouflages antigenic structures, such as β-glucan to avoid recognition by the immune system of the insect. Targeted disruption of *Mcl1* resulted in reduced virulence to *M. sexta* [29]. Since the *Mcl1* homolog *ClCE5* showed a necrotrophy-specific transcriptional activation, it may promote *in planta* fungal proliferation by inducing cell death, thereby facilitating fast growth of secondary hyphae that further kill and destroy plant tissues during the necrotrophic phase. Fasciclin proteins are GPI-linked cell surface proteins that mediate cell adhesion [40]. The biological function of fasciclin (*MoFLP1*) was demonstrated in *M. oryzae* wherein it is involved in conidiation, adhesion of fungal structures (conidia and appressoria) on the hydrophobic surface and virulence on rice [34]. It is likely that *C. lentis* secretes the fasciclin homolog ClCE8 during conidium and appressorium adhesion on the plant surface, though it might also mediate down-stream infection related to fungal development. Recently, a secreted laccase (*ClCE14* ortholog) was found to be up-regulated in appressoria formed *in planta* and during the biotrophic phase [3] supporting the role of fungal laccases during early fungal-plant interactions, and fungal virulence, as proposed for the chestnut blight fungus *Cryphonectria parasitica* [41]. However, the expression of *ClCE14* was found to be quite low throughout the *C. lentis* infection process.

A Toxin B homolog *ClToxB* was also identified in the cDNA library [5]. *Toxin B* is well characterized in necrotrophic pathosystems, such as *P. tritici-repentis* - wheat; however, its role in hemibiotrophic pathosystems, such as *C. lentis* - lentil remains unknown. Unlike *PtrToxB*, *ClToxB* is a single copy gene and no DNA polymorphism was found in *ClToxB* of the two isolates representing the two *C. lentis* races 0 and 1. However, expression levels varied with a higher transcript level observed for the virulent race 0 isolate compared to the race 1 isolate during the switch to necrotrophy (Fig. 8). Like PtrToxB (6.6 KDa), ClToxB (7.8 KDa) is likely a host (lentil)-specific toxin (Fig. 9). However, infiltration of the purified ClToxB in lentil tissues is required to confirm its role as a host-specific toxin. *Colletotrichum lentis* delivers the effector protein CtNUDIX into lentil cells specifically at the biotrophy-necrotrophy switch signaling a transition in the pathogen to the anthracnose causing necrotrophic phase by causing cell death [27]. ClToxB might be involved in amplifying cell death signals during the biotrophy-necrotrophy switch and thus may contribute to quantitative differences in virulence between the *C. lentis* races 0 and 1. To confirm the role of *ToxB* in *C. lentis* as a virulence factor, an RNAi approach was used to knock-down *ClToxB* mRNA levels. No growth or conidiogenesis penalty was observed among the silenced strains, which is consistent with observations in *P. tritici-repentis* that toxins have an exclusive role in pathogenesis. Three silenced strains displaying varying mRNA levels (5 to 80 % of the wild-type strain) were used to infect Eston plants. Severity of anthracnose disease was correlated with the *ClToxB* transcript level as SToxB-8 and SToxB-1 strains expressing *ToxB* at a rate of 5 and 80 % of the wild-type, respectively, caused significantly reduced anthracnose severity on leaves and stems (13.08 and 72.24 %, respectively compared to 87.5 % in plants infected with the wild-type isolate CT-21 at 6 dai) (Fig. 12b and c). Dot-plot analysis revealed a conserved syntenic block in the related species *C. higginisianum* and together with comparative phylogeny (species tree versus *ToxB* tree), GC pattern along the *ToxB* containing pseudomolecule, and codon usage profile *ToxB*, was confirmed not to be a foreign gene in *C. lentis* (Figs. 10 and 11). This is different from another, well studied fungal toxin gene, *ToxA*. This gene transferred from *S. nodorum* to the distantly related *P. tritici-repentis* possibly through conidial anastomosis, and conferred virulence to the latter enabling the fungus to become a serious pathogen on wheat where it causes tan spot disease [20]. This HGT was estimated to have occurred after 1941 because prior to that the tan spot pathogen was not considered a major threat to wheat production.

The transmission of genetic material through HGT is very common in bacteria, but has also been identified in several fungal species, particularly in terms of pathogenicity and virulence genes, resulting in pathogens broadening their host range and/or increasing their virulence. Such changes allow fungi to exploit new ecological niches, but may also result in resistance break-down in agricultural systems. Although the majority of suspected HGT events in fungi have a fungal gene donor, many acquired genes have also been traced back to bacteria, and a very few to plant species [19]. In the genus *Colletotrichum*, Jaramillo et al. [42] identified eleven HGT events from bacteria including the one encoding argininosuccinate lyase. Some of these HGTs are involved in niche adaptation and virulence. Mining the EST library of *C. lentis* for potential horizontally transferred genes identified the gene *Arg* of bacterial origin, previously found in several other *Colletotrichum* spp. [42]. The gene encodes argininosuccinate lyase and is involved in arginine biosynthesis, which is essential for fungal virulence [43]. Considering the high sequence identity with *Arg* in several bacterial species, it appears this gene was likely acquired from bacteria by a common ancestor of *Colletotrichum* species, and may have contributed to an expansion into or colonization of new niches and hosts by increasing virulence in the genus. Although homologs to this gene can be found in other fungal genera (e.g. *M. oryzae*, data not presented) indicating a potentially important role in fungi, sequence identity is very low with *Arg* from *Colletotrichum* species, suggesting a repeated introduction of this gene into fungal genomes from different bacterial species.

Candidate effectors identified in this study showed no sign of positive selection as no substitution mutation was found [ω (dN/dS) < 1], which is consistent with the view that partial resistance may not impose enough selection pressure on pathogen populations to allow for diversification and novel races to emerge [44]. The identified SNPs in candidate effectors *ClCE6* and *ClCE8* were silent, but polymorphisms were used to develop a race differentiation assay (KASPar assays). The *ClCE6* KASPar marker could differentiate race 0 isolates from those of race 1 (Fig. 4), suggesting that it may be co-segregating with the virulence governing locus/loci, hence can be used to determine the race identity of *C. lentis* isolates. Race indexing of *C. lentis* isolates is important not only for monitoring the population dynamics of the pathogen, but also for screening germplasm under field conditions. Sources of resistance to race 1 have been identified in the cultivated species *L. culinaris* and have been successfully introgressed into cultivars. High levels of resistance against the more virulent race 0 has only been found in some accessions in the secondary and tertiary gene pools [45, 46], and efforts are underway to incorporate race 0 resistance into cultivars.

## Conclusions

EST mining identified a set of *in planta* expressed candidate effectors. Comparative genomics of effectors revealed no sign of positive selection pressure at the intraspecific level, suggesting that *C. lentis* isolates are under stabilizing selection. Two synonymous SNPs were detected in two of the candidate effectors, one of which (ClCE6) allowed pathogenic race 0 isolates to be differentiated from race 1 isolates. EST mining and comparative genomics also identified the foreign gene *Arg* encoding argininosuccinate lyase from bacteria among ESTs, which was likely acquired by *Colletotrichum* from a bacterial species through HGT to improve/enhance virulence. In addition, *C. lentis* likely secretes a host specific toxin ClToxB in lentil cells during the biotrophy-necrotrophy switch to amplify cell death signals caused the effector ClNUDIX and contribute to quantitative differences in virulence between the races 0 and 1.

## Methods

### Bioinformatics

Previously, we constructed a biotrophy-necrotrophy switch-specific cDNA plasmid library from susceptible lentil cultivar Eston infected with *C. lentis* isolate CT-21 (race 1) [5]. In this study, 2000 new clones from this library were sequenced to identify candidate effectors. These ESTs were subjected to VecScreen (http://www.ncbi.nlm.nih.gov/VecScreen) to identify sequences belonging to the vector pBluescript II SK (+). Vector and adapter (GAATTCGGCACGGGAGG) sequences were manually trimmed, and the resulting EST sequences were queried against the NCBInr protein database using the BLASTX algorithm (http://www.ncbi.nlm.nih.gov/BLAST) and against the Consortium for the Functional Genomics of Microbial Eukaryotes EST database (http://cogeme.ex.ac.uk) using TBLASTX algorithm [47]. An ORF finder algorithm (http://www.ncbi.nlm.nih.gov/gorf) was employed to predict coding regions of candidate effectors in all six frames *ab initio*; the longest sequence with a stop codon preceded by an in frame ATG codon was translated into protein sequence. The amino acid sequence was then screened for potential SP and transmembrane helices using SignalP server version 4.1 with default settings (http://www.cbs.dtu.dk/services/SignalP) and TMHMM server version 2 (http://www.cbs.dtu.dk/services/TMHMM), respectively. Protein sequences containing putative SPs were queried against the NCBI non-redundant protein database using BLASP algorithm (http://www.ncbi.nlm.nih.gov/BLAST). *N*- and *O*-linked glycosylation sites were predicted using NetNGlyc 1.0 (http://www.cbs.dtu.dk/services/NetNGlyc ) and NetOGlyc 2.0 servers (http://www.cbs.dtu.dk/services/NetOGlyc ), respectively. Identified candidate effectors (Table 1) were mapped onto the *C. lentis* isolate CT-30 (race 0) draft genome (unpublished data) to identify single nucleotide polymorphisms (SNPs) using BioEdit sequence alignment editor [48]. In addition, we re-sequenced the full length cDNA of the previously identified *ClToxB* of *C. lentis*-infected lentil (GenBank Accession: JZ350031).

ClCE8 and ClToxB homologous protein sequences were aligned using Clustal W (Larkin *et al*. 2007) and ESPrint version 3.0 . MEGA 6 program [49] was used to compute evolutionary distance of ClCE8 homologs. Clustal W-aligned ToxB sequences were subjected to Seq2Logo [50] to generate sequence logo.

### Plant and fungal materials

Lentil plants of the Canadian cultivar Eston and compatible *C. lentis* isolate CT-21 were grown and routinely maintained as described previously [5]. CT-21 mycelia (vegetative hyphae) and ungerminated conidia were collected and flash-frozen in liquid nitrogen as described previously [5]. Lentil differential cultivar CDC Robin was used to differentiate isolates belonging to race 1 from race 0. Race 0 isolates are fully pathogenic on CDC Robin, which shows partial resistance to isolates of race 1.

### Infection time-course

Leaflets from 3-week-old lentil plants were detached and inoculated in Petri dishes lined with wet filter paper with droplets of CT-21 conidial suspension (5 × 10^4^ conidia mL^−1^). Inoculated leaflets were incubated with 12 h photoperiod. The progress of fungal infection was microscopically assessed. Infection sites were harvested using a 6 mm cork borer at 3 time points: Appressorium penetration phase [24 h after infection (hai)], biotrophic stage (48 hai, characterized by the presence of fat primary hyphae), and necrotrophic stage (72 hai, characterized by thin secondary hyphae). These leaflet discs were then flash-frozen in liquid nitrogen until required.

### Total RNA extraction and RT-qPCR

Total RNA from mycelia, ungerminated conidia and CT-21-infected lentil leaf tissues collected at 24, 48 and 72 hai was isolated using RNeasy Plant Mini kit (Qiagen, Hilden, Germany). After eliminating genomic DNA using RNase-free amplification grade DNase I (Invitrogen, Carlsbad, USA), total RNA (2 μg) was reverse transcribed in a 20 μL reaction volume using 200 U SuperScript reverse transcriptase (Invitrogen, Carlsbad, USA) following the protocol of the supplier. The resulting cDNA was diluted 10-fold in UltraPure DNase/RNase free-distilled water (Life Technologies, Cergy Pontoise, France).

Real-Time PCR detection platform CFX96 (Bio-Rad, Hercules, USA) was used to quantify the expression of candidate effectors. The actin gene was used as an endogenous control (reference gene). The 5 μL reaction contained 2.5 μL of 2X Fast SYBR Green Master mix (Applied Biosystems, Courtaboeuf, France), 200 nmol of each primer and 1 μL cDNA as template. The following thermal conditions were used to quantify the expression: 2 min of pre-heating at 95 °C followed by 40 cycles of 10 s at 95 °C and 30 s at 60 °C. For normalization, the threshold cycle (C_T_) values of the reference gene were subtracted from the corresponding C_T_ values of candidate effectors, generating ΔC_T_ values. The relative expression of candidate effectors was calculated by the comparative C_T_ method [51] using a ΔC_T_ value obtained for vegetative hyphae as a calibrator. All relative expression values of genes were reported as means ± standard errors of the means on a 2-log scale (Additional file 1). Average fold change values were summarized as a clustergram. Primers used in RT-qPCR analyses are listed in Additional file 2.

### SNP genotyping

Genomic DNA was extracted from mycelia of 52 *C. lentis* isolates (Table 2) using DNeasy Plant Mini Kit (Qiagen, Hilden, Germany). Two forward primers in which the 3-prime end corresponds to one of the SNP alleles (Allele C corresponds to race 0, T to race 1) and the 5-prime end is tailed with fluorophore FAM (C allele-specific primer) and VIC (T allele-specific primer), and a common reverse primer were used for each candidate effector, *ClCE6* and *ClCE8* (Additional file 3). KASPar SNP genotyping was performed on a CFX384 Real-Time System (Bio-Rad, Hercules, USA) following the protocol of the manufacturer (KBioscience, Hoddeston, UK). Percentage endpoint relative fluorescence was used to generate an allele discrimination plot. Race identity (0 or 1) of all 52 isolates based on SNP genotyping are listed in the Table 2.

### Phenotyping of *C. lentis* isolates

Lentil differential cultivar CDC Robin was inoculated in pathogenicity assays with various *C. lentis* isolates collected from Saskatchewan and Manitoba, Canada, to determine their race identity (Table 2). Lentil cultivar Eston susceptible to both races was used as a susceptible control. Phenotyping was conducted as described previously [7].

#### *ClToxB in planta* expression

The plasmid pGR106 used in the study was kindly provided by Dr. David Baulcombe (University of Cambridge, Cambridge, UK). Primers used to construct pGR106-ClToxB and pGR106-ClToxB∆SP vectors are listed in Additional file 4. The *ClToxB* open reading frame with and without SP (ClToxB∆SP) were cloned into pCR2.1 (Invitrogen, Carlsbad, USA) and sequenced. Confirmed DNA sequences were digested with the restriction enzymes *Cla*I and *Not*I and ligated into the potato virus X-based binary vector pGR106. Binary constructs were then used to transform *Agrobacterium tumefaciens* strain GV3101 carrying the helper plasmid pJIC Sa_Rep (pSoup). Infiltration assays with recombinant *A. tumefaciens* were performed on 4–6 weeks old tobacco (*Nicotiana tabacum*) as described previously [5]. The macroscopic phenotype was monitored from 2 to 10 dpi. Photographs were taken from 2 to 10 dpi.

### Phylogeny and comparative genomics

We retrieved ITS-5.8S, *GAPDH*, *CHS-1*, *HIS3*, *ACT*, *TUB2* and *ToxB* sequences from the NCBI GenBank database for *C. lentis*, *C. higginsianum*, *C. orbiculare*, *C. gloeosporiodes*, *C. fioriniae*, *Magnaporthe oryzae* and *P. tritici-repentis*. Phylogenetic analysis was conducted with the Bayesian inference (BI) method using MrBayes 3.1 [52]. The GTR + G + I nucleotide substitution model was used to infer the species tree based on the loci listed above and *ToxB* tree based on *ToxB* homologs. This analysis ran for 1 million generations using four Markov chain Monte Carlo chains (3 hot and 1 cold) and trees were sampled at every 100 generations. Twenty-five percent topologies were burned out to construct the consensus species and *ToxB* consensus trees. Neighbor Joining (NJ), Maximum Parsimony (MP) and Maximum Likelihood (ML) trees were constructed using MEGA6 [49].

*ClToxB* and *ChToxB*, and *ClArg* and *ChArg* loci containing scaffolds were retrieved from the draft genomes of *C. lentis* and *C. higginsianum* [3]. Comparative genomics was performed using MUMmer software package 3 [53]. The NUCmer function of MUMmer package was used to align scaffolds, and the MUMmerplot function was used to visualize the alignment as a dot plot.

We explored the possibility of HGT of *ToxB* in *C. lentis* using 4 criteria: Phylogenetic distribution, GC content of the pseudomolecule (chromosome), syntenic relationship between *ToxB* loci and codon usage [54]. A BI algorithm was used to generate a species tree from multiple loci, including ITS-5.8S, *GAPDH*, *CHS-1*, *HIS3*, *ACT* and *TUB2*, and a *ToxB*-based gene tree obtained from *C. lentis*, *C. higginsianum*, *C. orbiculare*, *C. gloeosporiodes*, *C. fioriniae*, *Magnaporthe oryzae* and *P. tritici-repentis*. Other algorithms, such as NJ, MP and ML resulted in nearly similar topologies of both, species and *ToxB* gene phylogenetic trees.

### *ClToxB* silencing and pathogenicity testing

The plasmid pSilent-Dual 1 [55] used in this study was kindly provided by Dr. Hitoshi Nakayashiki (Kobe University, Kobe, Japan). The *ClToxB* open reading frame was amplified using the primer ClToxBSF/ClToxBSR and cloned into the *Eco*RV site of the pSilent-Dual 1. Primers used in the study are listed in the Additional file 5. The plasmid contains two convergent RNA polymerase II promoters, Pgpd and PtrpC, from *Aspergillus nidulans*, thereby producing sense and antisense RNA pools of *ClToxB*. pSilent-Dual 1-ClToxB was used to transform *C. lentis* spheroplasts as described previously [27]. Resulting geneticin-resistant transformants were selected and subjected to RT-qPCR to quantify *ClToxB* expression level as described above. Three silenced strains were used for pathogenicity testing.

Twenty-one-day-old Eston plants were sprayed with 5 × 10^4^ conidia per mL (3 mL per plant) and anthracnose symptoms on leaves and stems were scored on a 0 to 10 scale (with 10 % increments) where 0 indicated no lesion and 10 was equivalent to 91-100 % disease severity. Isolates (CT-21 and silenced strains) were assigned to experimental units in an RCBD with 3 independent biological replications each consisting of 4 plants in one pot. Disease severity scores were collected at 6 dai from each plant of the three biological replications, values were transformed into % values using mid-class values and subjected to analysis of variance using the mixed model procedure (PROC MIXED) of SAS v.9.3 (SAS Institute, Cary, USA) to determine whether there were significant differences in disease severity scores between the strain with knock-down *ClToxB* mRNAs and the wild-type. Disease severity scores were reported as least squares means of % values ± standard error. The difference between treatments was determined by Fisher’s least significant difference (LSD, p ≤ 0.05).

### Availability of supporting data

All the supporting data are included as additional files with the online version of this article.

## Additional files

Additional file 1:
**RT-qPCR expression profiling of candidate effectors of** **Colletotrichum lentis.** Average C_T_, ΔC_T_, and 2^(− ΔC_T_), fold change and fold regulation values are listed in separate sheets. (XLS 37 kb) Additional file 2:
**RT-qPCR primers used to quantify expression of** **Colletotrichum lentis** **candidate effectors in an infection time-course on lentil cultivar Eston.** (XLSX 10 kb) Additional file 3:
**KASPar markers (ClCE6** **and ClCE8).** Two forward primers in which the 3-prime end corresponds to one of the SNP alleles (Allele C corresponds to race 0 and T to race 1) and the 5-prime end is tailed with fluorophore FAM (C allele) and VIC (T allele), and a common reverse primer were used for each candidate effector. (XLSX 9 kb) Additional file 4:
**Primers used in transient expression vector construction.** Primer set CT30ToxBF/R was used to clone *ClToxB* into the binary vector pGR106 whereas ClToxB without signal peptide was cloned into pGR106 using the primer set CT30ToxB∆SP/CT30ToxBR. (XLSX 9 kb) Additional file 5:
**Primers used to construct pSilent-Dual 1-ClToxB construct.** Underline sites are *Eco*RV restriction sites and used to clone *ClToxB* ORF into the plasmid pSilent-Dual 1. (XLSX 9 kb)
